# Morphology and transverse alignment of the patella have no effect on knee gait characteristics in healthy Chinese adults over the age of 40 years

**DOI:** 10.3389/fbioe.2024.1319602

**Published:** 2024-03-18

**Authors:** Zhengming Wang, Jiehang Lu, Haiya Ge, Zhengyan Li, Min Zhang, Fuwei Pan, Rui Wang, Hengkai Jin, Guangyue Yang, Zhibi Shen, Guoqing Du, Hongsheng Zhan

**Affiliations:** ^1^ Shi’s Center of Orthopedics and Traumatology, Shuguang Hospital Affiliated to Shanghai University of Traditional Chinese Medicine, Shanghai, China; ^2^ Institute of Traumatology and Orthopedics, Shanghai Academy of Traditional Chinese Medicine, Shanghai, China; ^3^ Department of Massage, Third Affiliated Hospital of Henan University of Chinese Medicine, Zhengzhou, China; ^4^ Shanghai University of Traditional Chinese Medicine, Shanghai, China; ^5^ The First Affiliated Hospital of Zhejiang Chinese Medical University (Zhejiang Provincial Hospital of Chinese Medicine), Hangzhou, Zhejiang, China

**Keywords:** morphology, alignment, patella, femur, tibia, gait

## Abstract

**Background:** The influence of patella morphology and horizontal alignment on knee joint kinematics and kinetics remains uncertain. This study aimed to assess patella morphology and transverse alignment in relation to knee kinetics and kinematics in individuals without knee conditions. A secondary objective was to investigate the impact of femur and tibia alignment and shape on knee gait within this population.

**Patients and methods:** We conducted a prospective collection of data, including full-leg anteroposterior and skyline X-ray views and three-dimensional gait data, from a cohort comprising 54 healthy individuals aged 40 years and older. Our study involved correlation and logistic regression analyses to examine the influence of patella, femur, and tibia morphology and alignment on knee gait.

**Results:** The patellar tilt angle or the patella index did not show any significant relationships with different aspects of gait in the knee joint, such as velocity, angle, or moment (*p* > 0.05, respectively). Using multivariate logistic regression analysis, we found that the tibiofemoral angle and the Q angle both had a significant effect on the adduction angle (OR = 1.330, 95%CI 1.033–1.711, *p* = 0.027; OR = 0.475, 95%*CI* 0.285–0.792, *p* = 0.04; respectively). The primary variable influencing the knee adduction moment was the tibiofemoral angle (OR = 1.526, 95% CI 1.125–2.069, *p* = 0.007).

**Conclusion:** In healthy Chinese individuals aged over 40, patella morphology and transverse alignment do not impact knee gait. However, the femoral-tibial angle has a big impact on the knee adduction moment.

## 1 Introduction

Anatomically, the knee joint, being the largest and most intricate joint in the body, comprises the femur, tibia, patella, and surrounding soft tissues, all working in concert to ensure knee stability within physiological limits (Zhao et al., 2021). Under normal physiological conditions, the knee joint capably fulfills its essential biomechanical functions. However, deviations in bone or soft tissue anatomy can lead to undesirable mechanical load distribution and persistent knee instability (Campos et al., 2022), ultimately paving the way for the development of knee osteoarthritis (KOA).

With the global population aging, the prevalence of KOA is estimated to be 22.9% in individuals aged 40 years and older, making it one of the leading contributors to chronic disability (Cui et al., 2020). Recognized risk factors encompass gender, genetic predisposition, obesity, aberrant gait biomechanics, joint laxity, and meniscal injuries (Heidari, 2011). Among these factors, the role of biomechanics in KOA development has consistently come to the forefront and been substantiated (Lee, 2014; Aljehani et al., 2022; Spierings et al., 2023).

Knee joint kinematics results from the intricate roto-translation movements characteristic of the tibiofemoral and patellofemoral articulations (Vasso et al., 2017). The patella functions as an articulating fulcrum, enhancing the moment arm of the extensor mechanism. Additionally, it enhances quadriceps muscle efficiency by elevating the extensor mechanisms away from the axis of rotation of the knee, thus increasing torque (Wheatley et al., 2021). Moreover, it assists in reducing frictional wear that could otherwise damage the extensor mechanism tendon. When the knee is fully flexed, the patella serves as a connection between the quadriceps and the patellar ligament. Daily activities subject the patellofemoral joint to compressive forces of 3.3 times body weight during stair climbing and 7.6 times body weight during squatting (Reilly and Martens, 1972). From 45° flexion to full extension, the patella articulates with the femur, displacing the extensor mechanism from the knee’s mechanical axis, thereby enhancing torque generation for terminal extension (LeBrun et al., 2012). Gait patterns vary based on the position and orientation of the patella relative to the trochlear groove and the alignment of the tibia in relation to the femur. Within the knee joint complex, the patella, the largest sesamoid bone in the body, plays a crucial role in knee biomechanics (Suzuki et al., 2013). Early intervention is believed to facilitate timely stabilization and restoration of knee biomechanics, thereby reducing the risk of concurrent knee pathology (Bornes et al., 2014). Consequently, a comprehensive investigation into the factors influencing knee joint biomechanics, early identification of biomechanical irregularities, and prompt intervention become crucial components in slowing the progression of KOA.

Currently, the assessment of KOA involves patient-reported outcome measures (PROMs) as well as various imaging techniques, including X-ray, computed tomography, magnetic resonance imaging, and gait analysis. While PROMs provide clinical insights, their subjective nature, potential ceiling effects, and reliance on pain rather than daily life activities limit their clinical value (Bolink et al., 2015). Imaging evaluation, on the other hand, is constrained by factors such as cost, time, accessibility, and the static nature of images (Vomer et al., 2023). Fortunately, recent advancements in three-dimensional motion analysis have enabled objective, quantitative, reproducible, and standardized evaluation of knee joint kinematics (Piriyaprasarth and Morris, 2007). Prior studies (Clark et al., 2016; Hösl et al., 2018; Murakami et al., 2018) have already established the influence of patellar sagittal and coronal alignment, as well as the mechanical axis, on knee joint kinematics.

As such, this study’s primary focus is to evaluate how patellar morphology and transverse alignment impact the kinetics and kinematics of the knee joint in individuals without pre-existing knee conditions. The secondary objective involves assessing the influence of femoral and tibial morphology, femoral-tibial angle (FTA), and Q angle on knee kinetics and kinematics within this specific population.

## 2 Materials and methods

### 2.1 Participants

This study received approval from the Ethics Committee, and written informed consent was obtained from all participants at our institution before their inclusion. A total of 54 eligible subjects (20 males and 34 females) were recruited from our hospital between December 2020 and December 2022. Exclusion criteria were as follows: 1) participants with a diagnosis of KOA (Zhang et al., 2010); 2) individuals younger than 40 years of age; 3) reported knee pain within the preceding year; 4) a history of lower limb injuries or knee surgery; 5) presence of tumors or tuberculosis; 6) any diseases or lower limb deformities that might impact gait patterns; 7) a Kellgren-Lawrence (K-L) radiographic disease severity scale score of grade II (Kellgren and Lawrence, 1957) or greater; 8) untreated medical conditions.

### 2.2 X-ray evaluation

Radiographs (AXIOM Aristos VX, Siemens, Germany) of the random knees of the participants were analyzed. Each subject’s knee was imaged from two distinct perspectives: a weight-bearing full-leg anteroposterior view and a skyline view. The KL radiographic grading system was employed, aligning with the criteria established by Farrokhi et al. (Farrokhi et al., 2015). Specifically, the KL grades were defined as follows: Grade 0 indicated the absence of osteophytes; Grade I denoted the presence of osteophytes approximately 1 mm in size, with an uncertain classification; Grade II signalled the presence of minimal osteophytes, along with potential joint space narrowing, cysts, and sclerosis; Grade III indicated moderate or definite osteophytes and/or moderate joint space narrowing; and Grade IV signified the presence of large osteophytes and/or severe joint space narrowing.

**FIGURE 1:**
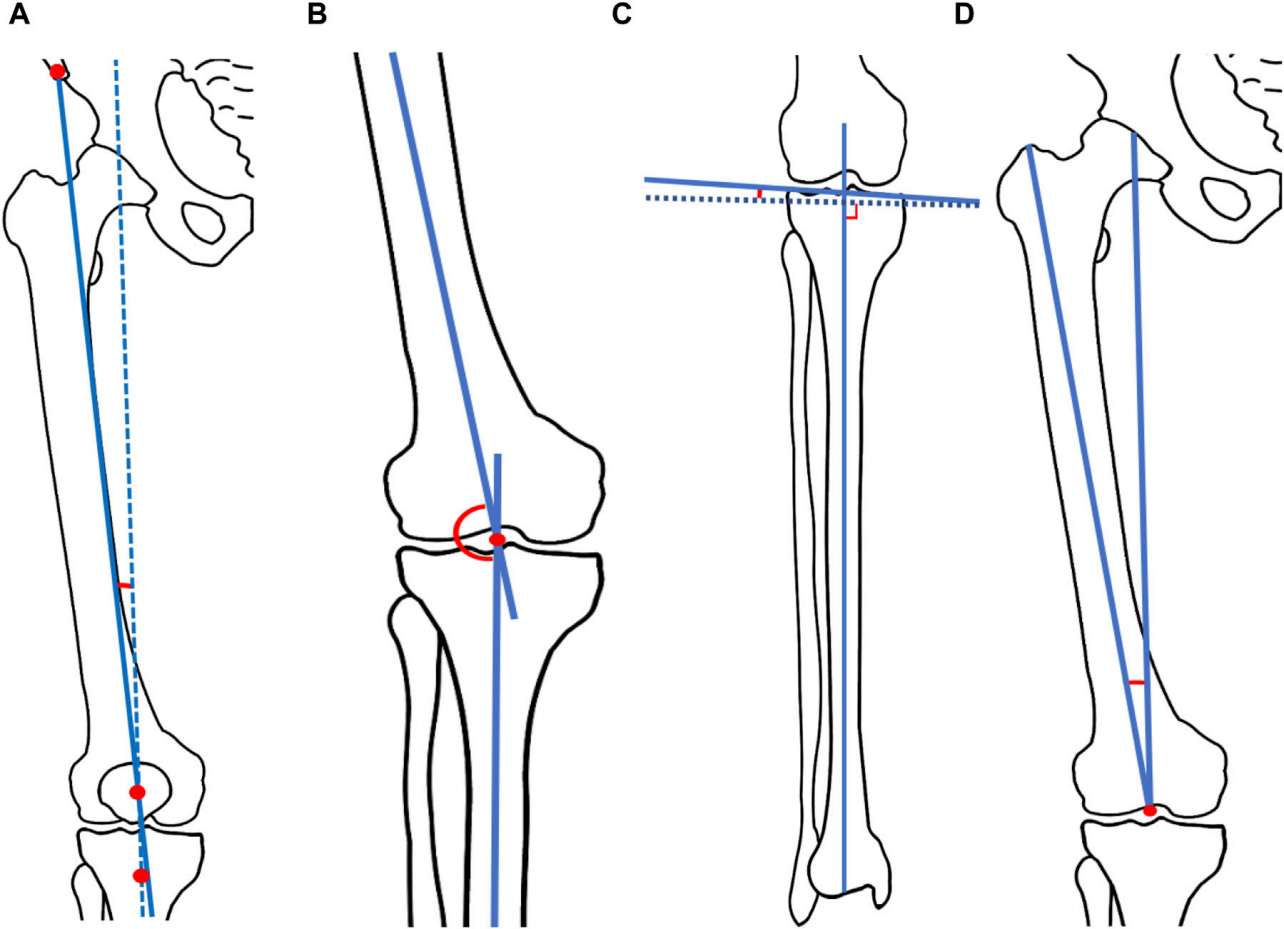
Schema of measured radiograph. **(A)** Q angle. The Q-angle represents the angle between two lines: the first drawn from the anterior-superior iliac spine to the mid-patella, and the second drawn from the mid-patella to the tibial tubercle. **(B)** Femoral-tibial angle (FTA). The FTA is defined as the lateral angle between the anatomical axes of the femur and tibia. **(C)** Proximal tibia varus angle (PTVA). The PTVA measures the angle between the tibial articular margins and a line perpendicular to the tibial mechanical axis. **(D)** Distal femoral valgus angle (DFVA). The DFVA represents the valgus angle between the femoral mechanical axis and the distal femoral anatomical axis.

Alignment and bony morphology were measured using established methods from prior studies (Chhabra et al., 2011; Stefanik et al., 2013). In the anteroposterior view, we assessed several angles (Dai et al., 2019; Kokubu et al., 2022; Hao et al., 2023; Milovanović et al., 2023), including the quadriceps angle (Q-angle), FTA, the proximal tibia varus angle (PTVA), and the distal femoral valgus angle (DFVA) (Figures 1A–D). The Q-angle represents the angle between two lines: the first drawn from the anterior-superior iliac spine to the mid-patella, and the second drawn from the mid-patella to the tibial tubercle. The FTA is defined as the lateral angle between the anatomical axes of the femur and tibia. The PTVA measures the angle between the tibial articular margins and a line perpendicular to the tibial mechanical axis. The DFVA represents the valgus angle between the femoral mechanical axis and the distal femoral anatomical axis.

In the skyline view, measurements were taken for the sulcus angle (SA) (Powers, 2000), patellar tilt angle (PTA) (Sasaki and Yagi, 1986), patella index (PI) (Cross and Waldrop, 1975), and trochlear depth (TD) (Damgacı et al., 2020) Figures 2A–D). The SA was defined by lines connecting the highest points of the medial and lateral condyles and the lowest point of the intercondylar sulcus. The PTA was defined as the angle subtended by the equatorial line of the patella and the line connecting the anterior limits of the femoral condyles. The PI, a guide to the understanding and diagnosis of patellofemoral instability, was calculated by the following method: two perpendicular lines were drawn, one through the maximum width and one through the maximum height of the patella, with their intersection point labelled “X.” The points where “X" met the lateral or medial cortex were labeled as “A" or “B.” PI was calculated as the ratio of (XB + AX) to (XB-AX) (Cross and Waldrop, 1975). TD represented the perpendicular distance from the line connecting the most anterior parts of the medial and lateral femoral trochlear facets to the deepest part of the trochlear groove (Damgacı et al., 2020). These radiographic measurements were performed by two experienced orthopedists using ImageJ software (Version 1.52, National Institutes of Health, United States) to derive the average values for continuous variables.

**FIGURE 2:**
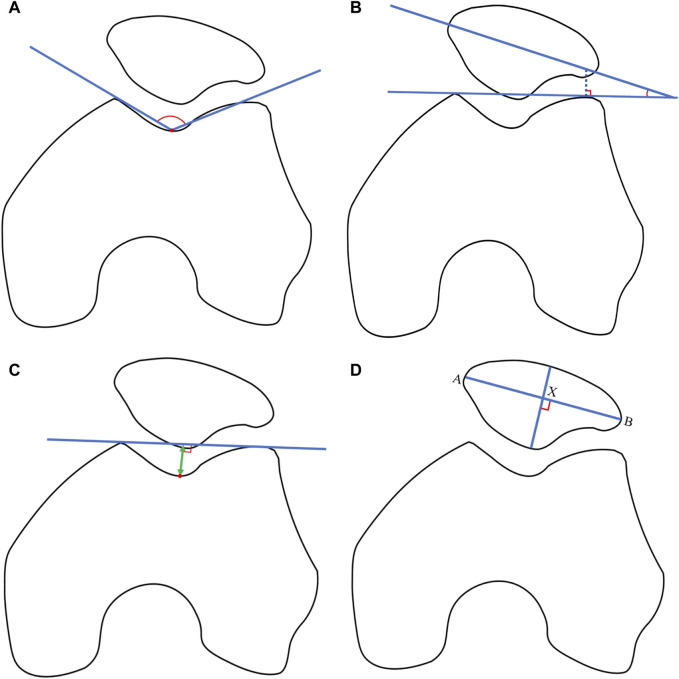
**(A)** Sulcus angle (SA). The SA was defined by lines connecting the highest points of the medial and lateral condyles and the lowest point of the intercondylar sulcus. **(B)** Patellar tilt angle (PTA). The PTA was defined as the angle subtended by the equatorial line of the patella and the line connecting the anterior limits of the femoral condyles. **(C)** Patella index (PI). The PI is were drawn by the two perpendicular lines, one through the maximum width and one through the maximum height of the patella, with their intersection point labelled “X.” The points where “X” met the lateral or medial cortex were labeled as “A” or “B.” PI was calculated as the ratio of (XB + AX) to (XB-AX). **(D)** Trochlear depth (TD). TD represented the perpendicular distance from the line connecting the most anterior parts of the medial and lateral femoral trochlear facets to the deepest part of the trochlear groove.

### 2.3 Gait analysis

Before each test, the instrument underwent calibration. The gait test followed the methodology detailed in our previous study (Zhang et al., 2022), offering a more comprehensive procedural explanation. In general, participants walked along an 8.6-meter-long and 6.5-meter-wide path, covered with timber and wooden flooring, at their self-selected pace. Motion data from retro-reflective markers were captured using the VICON motion capture and analysis system (VICON, Oxford, United Kingdom), operating at a sampling rate of 100 Hz and consisting of 16 cameras.

In line with a previous study (Riazati et al., 2022), quality assessments were performed using Visual 3D (Version 6.01.16, C-motion, United States) to eliminate excessively aberrant data, a crucial step in calculating kinematic and kinetic measurements (Jones et al., 2013). Both kinematic and analog data underwent filtration using a Butterworth 4th-order digital filter with cut-off frequencies set at 6 Hz for kinematics and 25 Hz for analog data. Gait speed was determined by dividing the distance walked by the time taken. Negative values are indicative of a particular direction. The external knee adduction moment (KAM) was normalized to each participant’s body mass. The initial KAM peak, representing the highest value within the first half of the stance phase, was assessed. Additionally, external knee moments for flexion, extension, external rotation, and internal rotation were evaluated.

### 2.4 Statistical analysis

All statistical analyses were performed using IBM SPSS Statistics version 16.0 (IBM, Armonk, NY, United States). The normality of continuous variables was assessed using Shapiro-Wilk tests. Normally distributed data were presented as mean ± standard deviation, while non-normally distributed data were summarized as median and interquartile range. Categorical data were expressed as counts (percentages). Spearman correlation analysis was employed to investigate the relationships between bone morphology, transverse alignment, and knee kinematic and kinetic outcomes. Generally, correlation coefficients less than 0.30 were considered indicative of weak correlations; those in the range of 0.30–0.50 were deemed moderate; and values exceeding 0.50 indicated strong correlations (McCaffrey et al., 2016). Variables with a *p*-value less than 0.1 in the Spearman correlation analysis were included in the regression model. For both univariate and multivariate logistic regression analyses, odds ratios (OR) were calculated after discretizing continuous variables based on their median values. The significance level was set at 0.05, and confidence intervals were reported at the 95% confidence level (CI).

## 3 Results

### 3.1 Participant characteristics

Table 1 presents the demographics and clinical characteristics of all study participants. The age of the participants ranged from 40 to 70, with a mean age of 54.3 ± 7.8 years. The body mass index (BMI) ranged from 17.2 to 29.9, with a mean BMI of 22.8 ± 3.1 kg/m^2^. The study included 20 male participants, and the majority had left knee joint involvement. Regarding bone alignment, the values for PTA, Q angle, and FTA were recorded as 2.0 (3.0), 12.9 ± 4.0, and 173.5 (4.3), respectively. In terms of knee joint bone morphology, the measurements for SA, patella index PI, TD, proximal PTVA, and DFVA were 133.0 (6.0), 6.8 (4.0), 4.8 (1.0), 3.0 (3.0), and 6.0 (1.0), respectively.

**TABLE 1:** Participant characteristics and gait indicators outcomes.

Variables	*N* = 54	Range	Variables	*N* = 54	Range
Age (years)	54.3 ± 7.8	40.0 to 70.0	Speed (m/s)	1.2 ± 0.1	0.9 to 1.4
BMI (kg/m^2^)	22.8 ± 3.1	17.2 to 29.9	Angle of the knee (°)		
Male (*n*/%)	20/37.0%		Flexion	68.3 ± 4.8	59.0 to 77.7
Left (*n*/%)	30/55.6%		Extension	3.3 ± 3.7	−3.2 to 12.3
Alignment (°)			Abduction	9.8 ± 5.5	−0.4 to 25.7
PTA	2.0 (3.0)	−6.9 to 7.0	Adduction	−1.7 (4.6)	−13.0 to 5.0
Q angle	12.9 ± 4.0	6.0 to 22.0	External rotation	−11.9 ± 4.9	−24.0 to −1.7
FTA	173.5 (4.3)	168.0 to 179.0	Internal rotation	3.8 ± 4.6	−8.4 to 14.4
Morphology			Moment of the knee (N·m/kg)		
PI	6.8 (4.0)	2.9 to 161.0	Flexion	0.6 ± 0.2	0.2 to 1.3
SA (°)	133.0 (6.0)	120.0 to 150.0	Extension	−0.3 ± 0.1	−0.6 to −0.2
TD (mm)	4.8 (1.0)	3.8 to 8.8	Adduction	0.4 ± 0.1	0.2 to 0.7
PTVA (°)	3.0 (3.0)	0 to 7.0	External rotation	−0.1 ± 0	−0.2 to 0
DFVA (°)	6.0 (1.0)	5.0 to 8.0	Internal rotation	0.2 ± 0	0 to 0.3

BMI, body mass index; PTA, patellar tilt angle; FTA, femoral-tibial angle; PI, patella index; SA, sulcus angle; TD, trochlear depth; PTVA, proximal tibia varus angle; DFVA, distal femoral valgus angle.

### 3.2 Kinematic and kinetic outcomes of knee motion

The walking speed of the participants ranged from 0.9 to 1.4 m/s, with an average speed of 1.2 ± 0.1 m/s. During locomotion, knee joint kinematics were as follows: flexion angle, 68.3 ± 4.8 degrees; extension angle, 3.3 ± 3.7 degrees; adduction angle, 1.7 (4.6) degrees; abduction angle, 9.8 ± 5.5 degrees; internal rotation angle, 3.8 ± 4.6 degrees; external rotation angle, 11.9 ± 4.9 degrees. Kinetic information during movement included the first external KAM peak, which was 0.4 ± 0.1 N m/kg. The external knee flexion and extension moments were 0.6 ± 0.2 and 0.3 ± 0.1 N m/kg, respectively. Additionally, the external knee external and internal rotation moments were 0.1 ± 0 and 0.2 ± 0, respectively. Detailed kinematic and kinetic outcomes are provided in Table 1.

### 3.3 Correlation between alignment or morphology and knee kinematics or kinetics

Correlation analysis revealed no significant relationship between speed and the alignment or morphological characteristics assessed in this study (*p* > 0.05, Table 2). The adduction angle showed moderate to strong correlations with age, gender, Q angle, FTA, and PTVA (r = −0.364, *p* = 0.007; r = 0.539, *p* < 0.001; r = 0.338, *p* = 0.012; r = −0.614, *p* < 0.001; r = −0.334, *p* = 0.014; respectively, Table 2). However, age was not found to be related to the kinetic parameters of the knee (*p* > 0.05, respectively). Notably, a robust positive association was observed between FTA and the first KAM peak in knee kinetics (r = 0.563, *p* < 0.001, Table 2). Moderate correlations were also noted between FTA and abduction angle, as well as PTVA and the first KAM peak (r = −0.388, *p* = 0.004; r = −0.303, *p* = 0.026; r = 0.362, *p* = 0.007; respectively). Weak correlations were found between BMI or PTVA and abduction angle, age and internal rotation angle, and gender and external knee extension moment (r = −0.278, *p* = 0.042; r = −0.287, *p* = 0.035; r = -0.299, *p* = 0.028; r = 0.295, *p* = 0.03; respectively, Table 3). Comprehensive illustrations of the correlations between alignment or morphology and knee kinematics or kinetics are provided in Tables 2, 3.

**TABLE 2:** Correlation of kinematics and kinetics with age, BMI, sex, PTA, Q angle, and FTA.

Variables	Age		BMI		Sex		PTA		Q angle		FTA	
	r	*P*	r	*P*	r	*P*	r	*P*	r	*P*	r	*P*
Speed	0.155	0.264	−0.121	0.385	−0.091	0.513	−0.157	0.256	0.040	0.774	0.211	0.126
Angle of the knee												
Flexion angle	0.106	0.445	0.208	0.131	0.128	0.357	0.017	0.905	−0.045	0.745	−0.063	0.653
Extension angle	0.238	0.083	−0.019	0.894	−0.007	0.958	−0.105	0.451	−0.099	0.475	0.020	0.883
Adduction angle	−0.364	**0.007**	−0.201	0.145	0.539	**<0.001**	−0.062	0.654	0.338	**0.012**	−0.614	**<0.001**
Abduction angle	−0.172	0.212	−0.278	**0.042**	0.231	0.092	−0.185	0.181	0.242	0.078	−0.388	**0.004**
External rotation	−0.245	0.074	−0.235	0.087	0.128	0.357	−0.052	0.708	0.231	0.089	−0.143	0.304
Internal rotation	−0.299	**0.028**	0.065	0.643	0.106	0.446	0.196	0.155	0.160	0.247	0.167	0.277
Moment of the knee												
Flexion moment	−0.016	0.909	−0.105	0.452	−0.015	0.916	0.051	0.714	−0.006	0.966	−0.055	0.619
Extension moment	−0.053	0.704	−0.009	0.948	0.295	**0.030**	−0.012	0.931	0.036	0.798	−0.226	0.100
Adduction moment^#^	0.201	0.145	−0.029	0.835	−0.303	**0.026**	0.009	0.946	−0.063	0.653	0.563	**<0.001**
External rotation moment	0.212	0.125	0.137	0.324	0.047	0.737	−0.028	0.839	0.033	0.814	−0.138	0.321
Internal rotation moment	0.197	0.154	−0.058	0.678	0.108	0.436	−0.055	0.694	−0.149	0.282	0.039	0.780

BMI, body mass index; PTA, patellar tilt angle; FTA, femoral-tibial angle. The boldface indicates *p* value < 0.05.

**TABLE 3:** Correlation of kinematics or kinetics with sulcus angle, patella index, trochlear depth, PTVA, and DFVA.

Variables	Sulcus angle		Patella index		Trochlear depth		PTVA		DFVA	
	r	*P*	r	*P*	r	*P*	r	*P*	r	*P*
Speed	−0.067	0.629	0.117	0.398	−0.024	0.866	−0.141	0.310	−0.166	0.229
Angle of the knee										
Flexion angle	0.008	0.955	0.121	0.382	−0.055	0.691	−0.131	0.343	−0.049	0.723
Extension angle	−0.054	0.699	−0.013	0.928	−0.057	0.682	0.025	0.860	−0.081	0.562
Adduction angle	−0.010	0.944	−0.109	0.434	−0.082	0.557	−0.334	**0.014**	0.219	0.111
Abduction angle	−0.057	0.684	0.004	0.977	−0.050	0.720	−0.287	**0.035**	0.238	0.083
External rotation	−0.104	0.452	−0.188	0.174	−0.044	0.749	−0.064	0.648	−0.033	0.814
Internal rotation	−0.014	0.921	−0.246	0.073	0.107	0.442	−0.225	0.101	0.080	0.565
Moment of the knee										
Flexion moment	−0.068	0.627	0.106	0.444	−0.009	0.951	−0.012	0.929	−0.032	0.818
Extension moment	−0.006	0.963	−0.159	0.250	−0.014	0.922	−0.190	0.168	0.092	0.510
Adduction moment	0.081	0.559	0.188	0.174	0.104	0.453	0.362	**0.007**	−0.156	0.259
External rotation moment	0.005	0.970	0.034	0.806	−0.077	0.578	−0.120	0.387	0.070	0.613
Internal rotation moment	−0.178	0.198	0.051	0.716	0.136	0.327	0.069	0.620	−0.159	0.251

PTVA, proximal tibia varus angle; DFVA, distal femoral valgus angle. The boldface indicates *p* value < 0.05.

### 3.4 Logistic regression analysis

Based on the correlation analysis results mentioned earlier, variables with a *p*-value less than 0.1 were incorporated into the regression model. In the univariate logistic regression analysis with the adduction angle as the dependent variable, independent variables included age, BMI, gender, Q angle, FTA, and PTVA (Table 4). Multivariate regression analysis findings revealed that both the Q angle and FTA had significant effects on the adduction angle, even after adjusting for other variables (OR = 1.330, 95% *CI* 1.033–1.711, *p* = 0.027; OR = 0.475, 95% *CI* 0.285–0.792, *p* = 0.04; respectively).

**TABLE 4:** Logistic regression analysis between adduction angle with demographics, Q angle, FTA, and PTVA.

Variables	Univariate analysis		Multivariate analysis	
	OR (95% CI)	*P*	OR (95% CI)	*P*
Age	0.893 (0.822–0.970)	**0.007**	0.895 (0.783–1.024)	0.106
BMI	0.960 (0.806–1.144)	0.650	1.057 (0.761–1.470)	0.739
Sex	0.120 (0.032–0.443)	**0.001**	0.540 (0.057–5.102)	0.591
Q angle	1.240 (1.053–1.460)	**0.010**	1.330 (1.033–1.711)	**0.027**
FTA	0.584 (0.435–0.784)	**< 0.001**	0.475 (0.285–0.792)	**0.004**
PTVA	0.704 (0.500–0.992)	**0.045**	1.281 (0.734–2.234)	0.383

BMI, body mass index; FTA, femoral-tibial angle; PTVA, proximal tibia varus angle; OR, odds ratios; CI, confidence level; Boldface indicates *p* value < 0.05.

Similarly, in univariate logistic regression analysis with KAM as the dependent variable, independent variables included age, BMI, gender, FTA, and PTVA. The results of the regression analysis demonstrated that FTA independently determined KAM, even after adjusting for other variables (OR = 1.526, 95% *CI* 1.125–2.069, *p* = 0.007, Table 5). In a univariate regression model, FTA and PTVA were observed as independent factors influencing the adduction angle (OR = 0.733, 95% *CI* 0.627–0.954, *p* = 0.016; OR = 0.683, 95% *CI* 0.482–0.967, *p* = 0.032; respectively). However, this influence became statistically insignificant after controlling for other variables, such as age, BMI, gender, Q angle, and DFVA (*p* > 0.05, respectively, Supplementary Table S1). The internal rotation angle of the knee did not exhibit any significant effect on age, BMI, gender, or PI in both univariate and multivariate regression models (*p* > 0.05, respectively, Supplementary Table S2).

**TABLE 5:** Logistic regression analysis between KAM with demographics, FTA, and PTVA.

Variables	Univariate analysis		Multivariate analysis	
	OR (95% CI)	*P*	OR (95% CI)	*P*
Age	1.021 (0.953–1.095)	0.551	0.977 (0.888–1.076)	0.643
BMI	0.916 (0.766–1.096)	0.338	0.865 (0.670–1.118)	0.865
Sex	2.653 (0.844–8.336)	0.095	2.602 (0.393–17.235)	0.321
FTA	1.578 (1.211–2.057)	**0.001**	1.526 (1.125–2.069)	**0.007**
PTVA	1.300 (0.936–1.807)	0.118	0.944 (0.608–1.464)	0.796

BMI, body mass index; FTA, femoral-tibial angle; PTVA, proximal tibia varus angle; OR, odds ratios; CI, confidence level; Boldface indicates *p* value < 0.05.

## 4 Discussion

The knee, a pivotal component of the locomotor system, serves as a weight-bearing joint susceptible to degenerative changes primarily driven by abnormal mechanical loads (Zhao et al., 2021; Campos et al., 2022). Therefore, it is imperative to acquire a comprehensive understanding of the multifaceted factors influencing the mechanical load conditions experienced by the knee joint. Such insights can inform the tailoring of prevention strategies to mitigate inherent risks. The geometric characteristics of the knee’s articular components play a pivotal role in predicting joint contact mechanics. There is a prevailing consensus that the biological function of the patella is intricately linked to the compatibility of the patellofemoral joint (PFJ), which, in turn, depends significantly on PFJ morphology and biological performance (Fox et al., 2012). Previous studies (Clark et al., 2016; Hösl et al., 2018; Murakami et al., 2018) have predominantly focused on the substantial roles played by sagittal and coronal alignments of the PFJ and the mechanical axis of the lower limbs in mediating knee joint mechanics. However, the impact of patellar morphology and transverse alignment remains an area that warrants more extensive investigation. Building on existing literature, this study is thus well justified.

Our study’s findings indicated that there was no statistically significant correlation between PTA, a measure of transverse alignment in the PFJ, or the PI, an indicator of patellar morphology, and knee movement kinematics or kinetics (*p* > 0.05, respectively). Notably, an increase in PTA and PI, which play a critical role in assessing patellar stability, may suggest patellar malalignment. Such malalignment has been associated with conditions such as patellar instability, patellofemoral pain syndrome, and chondromalacia patellae. In contrast to metrics like the tibial tubercle trochlear groove distance in the coronal plane and the Insall-Salvati ratio or Caton-Deschamps index in the sagittal plane (Verhulst et al., 2020), our distinct results underscore two key observations. On the one hand, significant variability in patellofemoral alignment suggests that the observed patellar features in our study population may not accurately represent the broader trends across all populations (Hochreiter et al., 2020). On the other hand, while patellar alignment in individuals with osteoarthritis may lead to morphological changes, subsequently affecting muscle function and mechanical loading, it is important to acknowledge that this perspective has yet to be confirmed in the general population.

Our study provided evidence supporting the idea that FTA exerted a substantial and independent influence on KAM in individuals without underlying knee conditions, even after adjusting for potential confounding factors (*p* = 0.007). The KAM, a structural risk factor for KOA, is frequently used as an indicator of medial-to-lateral knee joint load distribution during gait (Zhao et al., 2007). Previous studies (Miyazaki et al., 2002; Barrios et al., 2009) have found associations between static frontal plane knee alignment and peak KAM magnitude. Increased varus malalignment is believed to augment the knee ground reaction force lever arm, thereby increasing the KAM during gait and the risk of various knee-related conditions (Bennell et al., 2011).

Our correlation analysis revealed that among demographic factors, age, BMI, and gender did not have a statistically significant impact on the velocity of the healthy population. However, we did observe that age was associated with adduction and internal rotation angles. Additionally, BMI showed a sole association with the abduction angle, while gender was related to the adduction angle, extension moment, and KAM (*p* < 0.05, respectively). Nonetheless, the multivariate regression model did not demonstrate any significant disparities between demographic independent variables and dependent variables related to the adduction angle, or KAM. This suggested that the absence of discernible differences in dependent variables may be attributed to the unique characteristics of the normal population under investigation. Furthermore, differences in demographic factors can be linked to the Q angle and the FTA. Q angle is frequently cited as a possible predictor of knee pathology and lower limb injury (Rauh et al., 2007). The varus-valgus angulation of the knee has been associated with mediolateral distribution of loads on the knee’s articular structures. Minor increases in varus alignment (approximately 5%) have the potential to greatly increase (approximately 20%) medial knee loading (Schipplein and Andriacchi, 1991). Previous research (Tran et al., 2022) has shown variations in gender, age, and BMI concerning the Q angle and FTA, which in turn affect mechanical distribution by influencing moments, ultimately increasing the likelihood of developing KOA.

The consideration and design of therapeutic interventions aimed at reducing peak KAM are of paramount importance in the effort to delay or prevent the development and/or progression of KOA in middle-aged adults. Correcting varus alignment disperses high-pressure zones, resulting in significant improvements in knee pain and other KOA symptoms. Interventions such as footwear, knee bracing, exercise, and gait retraining may be appropriate for reducing varus tibial malalignment in KOA and should be periodically evaluated (Hatfield et al., 2016).

This study provides the initial comprehensive analysis of the relationship between knee imaging evaluation and three-dimensional gait analysis, enriching our understanding of how the morphology and transverse alignment of the patella, femur, and tibia impact knee kinematics and kinetics. However, it is essential to interpret the findings considering certain limitations. Firstly, our study exclusively recruited healthy senior adults, indicating the necessity for further investigations across diverse age demographics to thoroughly validate our findings. Secondly, it is worth noting that, compared to the sagittal plane, the frontal and transversal planes exhibit smaller ranges of motion. This characteristic increases the susceptibility of skin markers to soft tissue artifacts, thereby limiting the reliability of our results. Nevertheless, it is important to acknowledge that skin markers provide a non-invasive and reproducible method for visual motion capture. Thirdly, our study did not incorporate surface electromyography of the lower limb to evaluate muscle activity. Although it is improbable for healthy individuals to display abnormal muscle co-contraction, this aspect should be considered in future studies involving individuals with KOA. Finally, this study did not explore the correlation between patellar shape, alignment, and the onset of articular cartilage degeneration and KOA. Subsequent studies should include populations with KOA for comparative analysis and subsequent conclusions.

## 5 Conclusion

In summary, this study found that the morphology and transverse alignment of the patella do not significantly impact knee gait characteristics in a sample of healthy Chinese adults aged 40 years and older. Notably, the FTA emerges as a pivotal determinant influencing the KAM, a crucial parameter for assessing normal mechanical knee function. Future investigations should focus on establishing threshold values for FTA, Q-angle, and PTVA that could indicate an increased risk of mechanical dysfunction in individuals. Furthermore, there is a pressing need for comprehensive studies that encompass structural, passive, and dynamic elements to formulate treatments aimed at reducing the incidence of KOA.

## Data Availability

The raw data supporting the conclusion of this article will be made available by the authors, without undue reservation.

## Abbreviations

KOA, knee osteoarthritis; PROMs, patient-reported outcome measures; FTA, tibiofemoral angle; KL, Kellgren and Lawrence; PTVA, proximal tibia varus angle; DFVA, distal femoral valgus angle; SA, sulcus angle; PTA, patellar tilt angle; PI, patella index; TD, trochlear depth; KAM, knee adduction moment; OR, odds ratios; CI, confidence level; BMI, body mass index; PFJ, patellofemoral joint.
