# Supplementary material for: Morphology and transverse alignment of the patella have no effect on knee gait characteristics in healthy Chinese adults over the age of 40 years
Source: Front Bioeng Biotechnol. 2024 Mar 18;12:1319602. doi: 10.3389/fbioe.2024.1319602 (PMC10982314; doi:10.3389/fbioe.2024.1319602)
Supplement: Supplementary file 1 [file Table1.DOCX]

Supplementary table 1 Logistic regression analysis between abduction angle with demographics, Q angle, FTA, PTVA, and DFVA.

| Variables | Univariate analysis | | Multivariate analysis | |
| --- | --- | --- | --- | --- |
|  | OR (95% *CI*) | *P* | OR (95% *CI*) | *P* |
| Age | 0.983 (0.917-1.053) | 0.623 | 1.001 (0.912-1.098) | 0.991 |
| BMI | 0.928 (0.777-1.109) | 0.413 | 0.875 (0.686-1.117) | 0.283 |
| Sex | 0.526 (0.171-1.616) | 0.262 | 1.493 (0.256-8.708) | 0.656 |
| Q angle | 1.071 (0.934-1.227) | 0.327 | 0.996 (0.835-1.188) | 0.965 |
| FTA | 0.773 (0.627-0.954) | **0.016** | 0.789 (0.611-1.018) | 0.068 |
| PTVA | 0.683 (0.482-0.967) | **0.032** | 0.802 (0.527-1.219) | 0.302 |
| DFVA | 1.790 (0.735-4.360) | 0.200 | 1.556 (0.568-4.264) | 0.390 |

BMI, body mass index; FTA, femoral-tibial angle; PTVA, proximal tibia varus angle; DFVA, distal femoral valgus angle; OR, odds ratios; *CI*, confidence level; Boldface indicates *P* value <0.05.

Supplementary table 2 Logistic regression analysis between internal rotation angle with demographics and PI.

| Variables | Univariate analysis | | Multivariate analysis | |
| --- | --- | --- | --- | --- |
|  | OR (95% *CI*) | *P* | OR (95% *CI*) | *P* |
| Age | 0.945 (0.879-1.016) | 0.945 | 0.925 (0.845-1.012) | 0.090 |
| BMI | 0.989 (0.830-1.177) | 0.899 | 0.918 (0.741-1.139) | 0.438 |
| Sex | 1.0 (0.331-3.018) | 1.0 | 2.190 (0.465-10.316) | 0.322 |
| PI | 0.893 (0.771-1.035) | 0.133 | 0.877 (0.740-1.039) | 0.129 |

BMI, body mass index; PI, patella index; OR, odds ratios; *CI*, confidence level.
